# National audit of patient reported experience of radical cystectomy for bladder cancer pathways

**DOI:** 10.1002/bco2.422

**Published:** 2024-08-19

**Authors:** Preksha Kuppanda, Louisa Hermans, Alan Uren, Nikki Cotterill, Edward Rowe, Krishna Narahari, Andrew Dickinson, Jeannie Rigby, Jonathan Aning, C‐PAT Study Group (#) and the BAUS Section of Oncology, Edward Calleja, Matthew Perry, Mohamed Abdulmajed, Daben Dawam, Rustam Karanjia, Joseph John, Alexandra Colquhoun, Paramananthan Mariappan, Philip Goodall, Pete Cooke, Nikhil Vasdev, Richard Binney, James Catto, Paul Cleaveland, Oliver Fuge, Sanya Caratella, Benjamin Jackson, Vijay Sangar, Mohammad Zaynulabedin Miah, Arjun Nambiar, Francisco Lopez, Biral Patel, Matthew Simms, Justine Royle, Omer Altan, James Douglas, Amit Mevcha, Pragnitha Chitteti, Jo Cresswell, Rosie Blades, Omar al Kadhi, Vishwanath Hanchanale, Anthony Ta, Ashwin Sridhar, Jon Featherstone, Emmanuel Okpii

**Affiliations:** ^1^ Bristol Urological Institute, Southmead Hospital North Bristol NHS Trust Bristol UK; ^2^ British Association of Urological Surgeons London UK; ^3^ School of Health and Social Wellbeing University of the West of England London UK; ^4^ Department of Urology Cardiff and Vale University Health Board Cardiff UK; ^5^ Department of Urology University Hospitals Plymouth Plymouth UK; ^6^ Action Bladder Cancer UK Tetbury UK; ^7^ Population Health Sciences, Bristol Medical School University of Bristol Bristol UK; ^8^ East Sussex Healthcare NHS Trust East Sussex UK; ^9^ Royal Surrey NHS Foundation Trust Surrey UK; ^10^ Ysbyty Gwynedd Hospital Bangor UK; ^11^ Southend University Hospital Westcliff‐on‐Sea UK; ^12^ East Kent Hospitals University Foundation Trust Canterbury UK; ^13^ Royal Devon University Healthcare NHS Foundation Trust Exeter UK; ^14^ Cambridge University Hospitals NHS Foundation Trust Cambridge UK; ^15^ Western General Hospital Edinburgh UK; ^16^ Nottingham University Hospitals NHS Trust Nottingham UK; ^17^ The Royal Wolverhampton NHS Trust Wolverhampton UK; ^18^ East and North Hertfordshire NHS Trust Stevenage UK; ^19^ Sheffield Teaching Hospitals NHS Foundation Trust Sheffield UK; ^20^ Stockport NHS Foundation Trust Stockport UK; ^21^ University Hospitals Plymouth NHS Trust Plymouth UK; ^22^ University Hospitals of Leicester NHS Trust Leicester UK; ^23^ The Christie NHS Foundation Trust Manchester UK; ^24^ Manchester University NHS Foundation Trust Manchester UK; ^25^ Royal Berkshire NHS Foundation Trust Reading UK; ^26^ Newcastle Hospitals NHS Foundation Trust Newcastle UK; ^27^ Oxford University Hospitals NHS Foundation Trust Oxford UK; ^28^ Gloucestershire Hospitals NHS Foundation Trust Cheltenham UK; ^29^ Hull University Teaching Hospitals NHS Trust Hull UK; ^30^ Aberdeen Royal Infirmary Aberdeen UK; ^31^ University Hospitals Coventry and Warwickshire NHS Trust Coventry UK; ^32^ University Hospital Southampton NHS Foundation Trust Southampton UK; ^33^ University Hospitals Dorset NHS Foundation Trust Bournemouth UK; ^34^ South Tees Hospitals NHS Foundation Trust Middlesbrough UK; ^35^ Lancashire Teaching Hospitals NHS Foundation Trust Preston UK; ^36^ Norfolk and Norwich University Hospitals NHS Foundation Trust Norwich UK; ^37^ Liverpool University Hospitals NHS Foundation Trust Liverpool UK; ^38^ University College London Hospitals NHS Foundation Trust London UK

**Keywords:** bladder cancer, cystectomy, multicentre outcome audit, PREM, PROM

## Abstract

**Objective:**

The objective of this study was to measure and describe the national patient experience of radical cystectomy (RC) pathways in the UK using the validated Cystectomy‐Pathway Assessment Tool (C‐PAT).

**Patients and Methods:**

A cohort of 1081 patients who underwent RC for bladder cancer, between 1 January 2021 and 31 July 2022 at 33 UK cystectomy centres, returned completed C‐PAT responses. SPSS was employed for data summary statistics, including median, interquartile range, Mann Whitney U test or Chi‐square test with a 95% confidence interval to assess statistical significance between potentially associated variables. Open‐text responses in the C‐PAT tool were analysed and coded using NVivo software.

**Results:**

In this cohort, the greatest perceived delay in the RC pathway, reported by 19% of patients (*n* = 208), was at the GP consultation to first hospital referral stage with suspected bladder cancer. Around 10% of patients perceived delays at each of the other stages in their pathway. Cancer nurse specialist (CNS) contact was strongly associated with an improved patient experience (*p* < 0.001); however, 9.5% of patients reported that they were not assigned a cancer nurse specialist in their pathway. Overall, 96% (*n* = 1028) reported their experience of RC pathway care to be good or excellent. There were no significant differences in reported patient experience found between cystectomy centres.

**Conclusion:**

This audit demonstrates the feasibility of measuring patient experience of RC pathways at scale. The C‐PAT tool demonstrated utility in identifying specific pathway areas for quality improvement. Overall UK patients report a high quality pathway experience. A focus on improving the referral pathway between primary and secondary care is necessary.

## 1 INTRODUCTION

High risk bladder cancer has lethal potential. Muscle invasive bladder cancer is associated with a 5‐year survival rate of 55%, and T1 high grade disease has been shown to have a 5‐year disease specific survival rate of 80–90%.^1^ Radical cystectomy (RC) is recommended as a treatment option for these patients and those with recurrent high grade non‐muscle invasive bladder cancer.

RC and urinary diversion may be associated with complications that impact on quality of life.^2^, ^3^, ^4^, ^5^ The RC pathway and multidisciplinary team interactions at stages from diagnosis to aftercare vitally contribute towards patient outcomes and experience. Most existing literature is framed from a clinical viewpoint with limited large scale holistic investigation of patients' perceptions or experience of their RC pathway.^6^ There is an urgent need to address this gap in knowledge. Patient experiences and satisfaction form a critical component of the quality assessment of any pathway and provide real‐world information to support quality improvement, clinical effectiveness and patient safety.^7^

The validated Cystectomy‐Pathway Assessment Tool (C‐PAT) is a patient completed tool that measures quality of care within the cystectomy patient pathway.^8^ In the United Kingdom (UK), RC for bladder cancer has been centralized to high volume centres.^9^ Approximately 2100 RCs are performed for bladder cancer each year in England.^10^ This study aimed to audit national patient experience of their RC pathway using C‐PAT.

## 2 PATIENTS AND METHODS

### 2.1 Study design and setting

**FIGURE 1:**
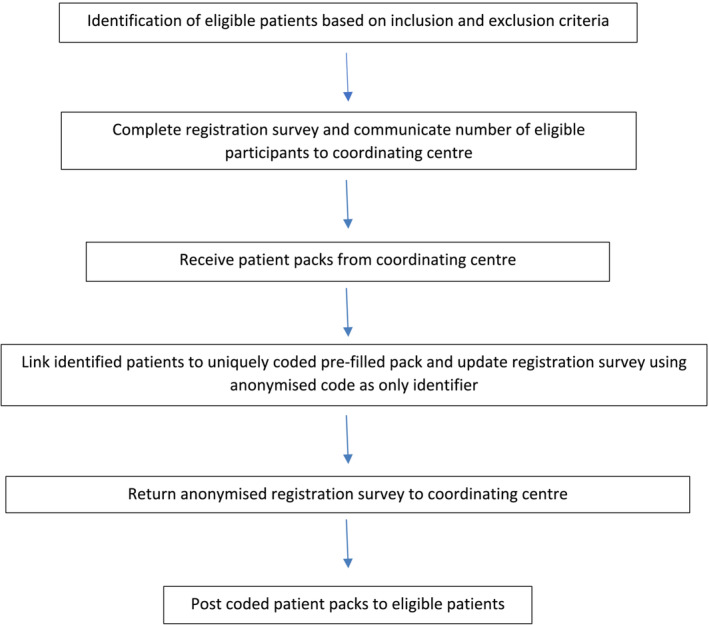
Flow diagram of the study process for each centre.

A ‘snapshot’ audit was designed to investigate UK patient experience of their RC pathway. All centres (n = 49) across the UK performing RC were contacted and invited to participate by the British Association of Urological Surgeons (BAUS), Section of Oncology. Centres that expressed an interest in participating were contacted in advance of commencing the audit to ensure that the audit received institutional approval before starting and to advise of the codes needed to identify patients. The audit was conducted between 14 November 2022 and 21 June 2023. The flow diagram in Figure 1 illustrates the study design.

### 2.2 Participants

Participating centres were invited to retrospectively identify all patients undergoing RC for bladder cancer between 1 January 2021 and 31 July 2022. Centres completed an anonymous, centre level, registration survey describing the number of potentially eligible participants, demographic details and pathway dates. Patients alive during the survey period were eligible to be included in the audit and invited to complete a postal C‐PAT questionnaire.

### 2.3 C‐PAT survey methodology

Participating centres were sent pre‐prepared patient packs that corresponded with their registration survey response. Each pack included a patient letter explaining the purpose of the audit, a C‐PAT questionnaire and a pre‐paid envelope to enable return of the questionnaire directly to the coordinating centre. Each C‐PAT questionnaire was uniquely coded to enable the coordinating centre to determine the number of responses from each participating centre and allow anonymous comparison of patient responses with centre provided demographic data. Patient records and identifiable data were accessed only by the directly responsible clinical care team at each institution. Each participating centre was responsible for posting the patient packs to their own patient cohort. No reminder or follow up correspondence was sent to patients who did not return the questionnaires. All C‐PAT received at the North Bristol NHS Trust coordinating centre were anonymous and uploaded into a securely hosted REDCAP database.

### 2.4 C‐PAT questionnaire

The C‐PAT^8^ is a 17‐item questionnaire with a five‐item scoring domain for ‘care and support’, scored between 0 and 16, with higher scores indicating greater care and support. The tool captures surgical demographics, operational factors such as delays within the pathway, patient experience during treatment and surgery, and patient perceived complications post‐operation. Two free‐text boxes are also provided to enable patients to provide additional comments and feedback. The C‐PAT questionnaire is illustrated in Figure S1.

### 2.5 Data and analysis

The Statistical Package for the Social Sciences (SPSS Inc, IBM Corp., Armonk, NY, United States) was used to perform summary statistics on the data. This involved calculating median, interquartile range and performing Mann Whitney U test or Chi‐square test with 95% confidence interval to determine statistical significance between potentially associated variables. The open‐text responses within the C‐PAT tool were subjected to analysis and coded using the NVivo software.

## 3 RESULTS

### 3.1 Demographics

In total, 33/49 (67%) UK centres participated in the C‐PAT audit (see Table S1). From these centres, 1714/2093 (81.8%) patients were eligible for inclusion at the snapshot timepoint. Complete C‐PAT responses were received from 1081/1714 (63%) patients; 779/1081 (75.3%) were male, and the median age of respondents at RC was 71 years. The route to diagnosis for most respondents, 585/1081 (59.9%), was the Two Week Wait (2ww) pathway. The 2ww pathway describes a UK pathway for GPs to refer patients with suspected cancer to see a specialist within 14 days with the aim of shortening the time to diagnosis and treatment. Centre provided data demonstrated the median time from 2ww referral to undergoing RC for patients with muscle invasive bladder cancer who underwent neo‐adjuvant chemotherapy was 162 days (IQR: 136–209) versus 102 days (IQR: 73–130) for those who did not versus 112 days (IQR: 52–222) for patients referred from non‐2ww pathways. Robotic RC was performed in 631/1081 (61.1%), and an ileal conduit urinary diversion was performed in 982/1081 (95.1%). Table 1 summarizes the demographic of the audit population.

**TABLE 1:** Demographic of C‐PAT respondents (*n* = 1081).

Demographic variable	*n* (%)
Median age of patient at cystectomy	71
Gender, *n* (%)	
Male	779 (75.3)
Female	255 (24.7)
Salvage cystectomy, *n* (%)	51 (4.9)
Neo‐adjuvant chemotherapy, *n* (%)	276 (26.7)
Nephrostomy/JJ stent in situ at the time of cystectomy, *n* (%)	178 (17.8)
Surgical method for performing cystectomy, *n* (%)	
Open	398 (38.5)
Laparoscopic	4 (0.4)
Robotic	631 (61.1)
Urinary diversion	
Conduit	982 (95.1)
Neobladder	35 (3.4)
Other diversion	16 (1.5)
Source of first referral for bladder cancer, *n* (%)	
2ww^a^	585 (59.9)
Emergency admission	44 (4.5)
Already under surveillance for upper tract urothelial carcinoma (UTUC)	68 (7.0)
Other referral pathways^b^	280 (28.7)
Diagnosis leading to cystectomy	
Muscle Invasive Bladder Cancer (MIBC)	554 (54.3)
Non‐Muscle Invasive Bladder Cancer (NMIBC)	314 (30.8)
Recurrent NMIBC	152 (14.9)

^a^ The 2 week wait GP referral system allows a patient with symptoms that may indicate an underlying cancer to be seen as quickly as possible.

^b^ Other specialty referral, emergency referral, others.

### 3.2 Patient perceptions of pathway delays from diagnosis to radical cystectomy

C‐PAT captured patient perceived delays at different stages of their pathway. Most patient perceived delays were surrounding the initial GP decision to refer to the hospital phase of their pathway with a median of 19.2% of patients at each centre reporting this (IQR: 15.3–23.5). Reasons for the delay given by respondents were that their symptoms were overlooked or misdiagnosed:

I was given antibiotics for a UTI‐ twice! Because of my age … The only thing I'd change if I could is, I'd make all GPs aware that women can get bladder cancer in their 30s ‐ it's not always a UTI. They actually told me it definitely wasn't cancer!

It was nearly 12 months from my first GP appointment with blood in my urine (but no infection present) until my GP finally referred me to urology. I am certain that the delay meant my outcome was worse than it could have been.

Delays were perceived by patients in subsequent stages of their pathway from diagnosis to undergoing RC and these are illustrated in Table 2. There was a trend that more patients perceived a delay in their pathway if they underwent their diagnostic TURBT and RC at different hospitals but no statistically significant difference (*p* = 0.873) was found when this group was compared with patients receiving their TURBT and RC at the same centre.

**TABLE 2:** Centre reported timings to cystectomy and patient perception of pathway delays.

Time/days passed within each stage of the pathway (centre reported data)		
Referral to cystectomy; n, median days (IQR)	843, 156 (94–286)	
2ww	545, 164 (105–281)	p‐value < 0.001
Other pathways	199, 112 (52–222)	
**Referral to TURBT; *n*, median days (IQR)**	629, 38 (26–63)	
2ww	485, 37 (27–56)	p‐value = 0.549
Other pathways	158, 40.5 (21.75–115.5)	
**TURBT to Cystectomy; *n*, median days (IQR)**	860, 127 (71–250.75)	
2ww	548, 126 (72–222.75)	p‐value = 0.277
Other pathways	286, 125 (71–270)	
Same hospital	380, 134 (71.25–257.75)	p‐value = 0.433
Different hospital	478, 117 (71–237.75)	
Patients who reported delay from decision to remove bladder to surgery on the C‐PAT tool	76, 125.5 (71.25–341.5)	p‐value = 0.631
Patients who did not report delay from decision to remove bladder to surgery on the C‐PAT tool	772, 127 (72–250.75)	
**C‐PAT reported delays, *N* = 1081**		
GP consultation to first hospital referral; n, median % (IQR)	189, 19.2 (15.3–23.5)	
Proportion from those who had initial diagnosis/tests and surgery at different hospitals, *n* (%)	125 (66.1)	
GP referral to hospital visit; *n*, median % (IQR)	97, 10.1 (5.1–14.7)	
Proportion from those who had initial diagnosis/tests and surgery at different hospitals, *n* (%)	64 (65.9)	
First test and scan to receiving results; *n*, median % (IQR)	108, 8.7 (4.5–12)	
Proportion from those who had initial diagnosis/tests and surgery at different hospitals, *n* (%)	70 (64.8)	
Bladder removal decision to surgery; *n*, median % (IQR)	110, 7.7 (3.7–13.1)	
Proportion from those who had initial diagnosis/tests and surgery at different hospitals, *n* (%)	65 (59)	

*Note*: IQR represents inter quartile range across centres.

### 3.3 Patient perceptions of post‐radical cystectomy management pathway

Patient reported post‐operative complications were strongly associated (*p* < 0.001) with patient perceived experience of their overall care. In total 12.4% (IQR: 6.7–16.7) reported complications in the first 3 months after their RC that required further treatment with a local or general anaesthetic. Most patients reported positive experiences and meeting of their post‐operation expectations, with 86.4% reporting that they felt prepared for what to expect after surgery. Furthermore, a median of 92.3% perceived that they had timely follow‐up and contact post discharge and 86.4% reported receiving sufficient support to manage their stoma. Data captured on post operative complications, support and follow‐up experience are summarized in Table 3. Poor after care was described by a small proportion of patients. Some felt they did not receive enough information and others that their follow‐up schedule was sub‐optimal after surgery:

**TABLE 3:** Patient perceptions of post radical cystectomy support and follow up *N* = 1081.

	*n*; median (IQR)
Felt prepared for what to expect after operation	901; 86.4 (80–92)
Had timely contact after discharge to discuss any future follow up	935; 92.3 (87.9–96)
Received adequate support to manage new stoma or new bladder after discharge	912; 86.4 (82.7–92.1)

I was given lots of info pre‐op however there wasn't enough information post‐op regarding hernia and how to avoid.

I was not advised about some of the post‐operative complication e.g., lymphoedema.

The system let me as a patient down, in the after care. Post‐surgery I feel abandoned, and my mental health has been affected.

I am supposed to have a 6 monthly scan, but none have been forthcoming which is causing me great concern.

It will be 2 years in August since my bladder removal. In that time, I have only seen the consultant once. I was told I would need a scan every 6 months, this has not happened.

The care was good, but my follow up appointment was cancelled four times. So instead of 4–6 weeks as stated, it was three months before I got seen, which caused me great distress.

### 3.4 Patient experience with access to key Allied Health Professionals during their pathway

In total, 101/1081 (9.5%) patients reported that they were not assigned a cancer nurse specialist (CNS) and did not receive support from a CNS at all during their cystectomy pathway. Whilst the remainder had received the contact details of a CNS, 7.6% (81/1081) indicated that it was difficult to contact their respective CNS. Chi‐square test demonstrated a strong association between CNS contact and patient reported experience of care (*p* < 0.001). Some qualitative responses acknowledged and appreciated the role of CNS in their care pathway; one such comment was

Initially the cancer diagnosis was a real shock, and I was referred to [anonymized] Hospital but the cancer nurse specialist was excellent at explaining the referral and the actual surgery.

Stoma care was an area where some patients voiced the need for increased support and more access to stoma nurses:

The only negative comment I would make is I have had very little contact with local stoma nurses not even an odd phone call to see how things are or if I have any queries.

Stoma nurses were very rushed so had no time for extra questions or to go over procedure twice, which would have been helpful as I am 79 years old.

The stoma nurses are key. There need to be enough of them, adequately funded.

### 3.5 Overall patient experience of the cystectomy pathway

Most patients, 1028/1081 (96%), reported their overall care experience to be positive (excellent, 71.5%; good, 24.5%). Table 4 outlines the C‐PAT results for patient experience. C‐PAT examined the experience of the component parts of the RC pathway. The patient reported experience of their hospital stay for RC and care received during their RC pathway for the cohort was overall positive with 776/1081 (72.9%) reporting receiving excellent care, followed by 227/1081 (21.3%) reporting good care. Most patients, 845/1081 (79%) reported that they were extremely likely to recommend the hospital(s) where they underwent investigation and RC to friends/family with similar problems. Chi‐square test demonstrated that patient reported experience of care received was not significantly influenced or associated with patient perceived delays such as delay in receiving results after initial tests and scans or delay in receiving surgery after a decision was made to undergo RC. Additionally, no significant association was noted between patient reported experience and change in hospital for initial tests and final surgery.

**TABLE 4:** Patient experience. STATEMENT

Rating for the care received whilst in hospital for the operation to remove your bladder, n (%)	*N* = 1065
Excellent	776 (72.9)
Good	227 (21.3)
Average	46 (4.3)
Poor	13 (1.2)
Extremely poor	3 (0.3)

Likeliness to recommend the hospital where the cystectomy was performed, n (%)	*N* = 1070
Extremely likely	845 (79)
Likely	172 (16.1)
Neither likely nor unlikely	34 (3.2)
Unlikely	8 (0.7)
Extremely unlikely	11 (1)

Rating for overall care received, *n* (%)	*N* = 1071
Excellent	766 (71.5)
Good	262 (24.5)
Average	26 (2.4)
Poor	14 (1.3)
Extremely poor	3 (0.3)

The median score for the ‘care and support’ domain was 16 (IQR 14–16), which is the highest score associated with the respective domain. No statistically significant difference between scores were noted between the centres (*p* = 0.467).

## 4 DISCUSSION

This national audit is the first to comprehensively assess patient reported experience of the entire RC pathway at scale. We have shown that across a health system overall patient experience of the pathway is above average for over 95% of patients. C‐PAT demonstrated utility in identifying specific areas for pathway improvement, highlighting where patients were not able to access adequate CNS and Stoma support in addition to circumstances where follow‐up post‐surgery had been sub‐optimal. This study found that around 20% of respondents felt that their referral to secondary care was delayed when they presented to their General Practitioner (GP) in the community and that their presenting symptoms were overlooked or misdiagnosed.

Delay in RC after diagnosis has been associated with detrimental effects on survival; however, heterogeneity in how delay is defined has been evidenced.^11^ In our study, the median time to RC was 156 days, highlighting no significant difference in time to RC in this contemporary cohort compared to previous UK studies.^12^, ^13^ Additionally, we have shown no difference in pathway length between patients undergoing RC at a different hospital to where their TURBT was performed and those receiving all procedures in one institution. These data highlight that whilst surgeons may concentrate on performing high quality surgery, there is a need to focus on shortening the patient pathway before RC. Importantly, the C‐PAT data inform us that our patient cohort did not perceive a significant delay in their RC pathway. Crucially, this implies that whilst healthcare professionals continue to quality improve the pathway it should be considered whether a piece of work to raise health awareness for patients regarding on pathway timing is needed. Worryingly, we found patients' perception of delay in their pathway was mainly related to the time between presentation to their GP and referral to secondary care. This novel but important finding reinforces the findings of a smaller mixed methods study^14^ and demonstrates that barriers and missed opportunities for referral need to be investigated as this is a hidden aspect of patients bladder cancer pathway, which may contribute to existing survival outcomes.^14^ Though it is essential to acknowledge that delays during the GP referral phase may be influenced by a variety of factors,^15^ there is a need to urgently explore this finding further to improve this vital part of the pathway for bladder cancer patients.

C‐PAT was designed to concisely measure aspects of quality of care within the RC pathway. In this cohort the tool identified when access to CNSs, stoma care support and follow‐up were suboptimal. CNSs contribute to improving the outcomes for individuals with cancer.^16^, ^17^ In the UK, access to a CNS is a National Institute for Health and Care Excellence (NICE) quality standard.^18^ In real‐life practice, we have shown that a significant minority of around 10% did not receive contact from a CNS and a further 7.6% received contact details but found it hard to contact their CNS. Our findings add detail to previous National Cancer Patient Experience Survey data indicating that bladder cancer patients are underserved by CNS contact compared to other patients with cancer.^19^ Furthermore, our C‐PAT findings reinforce the positive effect that CNS contact has on patients experience of their RC pathway. Stoma care support and follow‐up form an essential part of post RC aftercare; 13.9% of patients reported dissatisfaction with this phase of the pathway and were able to highlight the need for increased stoma support in addition to missed follow‐up using C‐PAT. Patients were also able to self‐report their experience of complications. Present C‐PAT evidence has given patients a voice to inform future patient experiences. Further research into the longer term functional and emotional impact of radical cystectomy is needed. Centre specific data from this audit have been shared with each participating centre for reflection and will be used to guide quality improvement where necessary. The C‐PAT provides a standardized tool to monitor the result of any quality improvement at a centre level.

Overall, our national audit has reassuringly demonstrated that most patients surveyed regarded their experience of their RC pathway as more than satisfactory. C‐PAT captured the highest median score for patient experience within the ‘care and support’ domain and participating centres should be praised for this. Our recommendation is that centre specific information is regularly monitored and shared locally with patients and referral networks. In this cohort of patients, C‐PAT did not show any statistically significant difference in the quality of care received on pathways between cystectomy centres. This finding is in line with the results of Uren et al.,^8^ and this likely suggests that there may not be significant differences in pathways between centres presently in the UK. We acknowledge though that 15/49 centres did not participate. Further use of the C‐PAT internationally would be valuable to triangulate whether there are opportunities to learn from others.

The present study is not without limitation; it is essential to note that no case‐adjustments, including receipt of chemotherapy, were made to compensate for the varying number of patient responses across participating centres. C‐PAT may not be sensitive enough to determine small differences between centre pathways however the purpose of the instrument is to guide the provision of quality of care for patients assessing key aspects important to patients and clinicians rather than between centre evaluation. This audit comprised a contemporary population, though the study period was after the main waves of COVID‐19; the impact of COVID‐19 on the patient reported experience in this study is unknown. This audit had a high response rate but the authors acknowledge nonresponse rates are a potential source of bias because the experience of the non‐responders is unknown. The strength of the present study though is that it provides information on the patient experience of the RC pathway in a large, unselected cohort of patients and provides a benchmark for current UK practice.

## 5 CONCLUSION

This national snapshot audit provides unique and invaluable insight into the patient experience of RC for bladder cancer treatment pathways. Overall, patients report a high‐quality pathway experience. C‐PAT has demonstrated utility in identifying specific areas for improvement in the patient pathway.

## AUTHOR CONTRIBUTIONS

Preksha Kuppanda and Jonathan Aning had full access to all the data in the study and take full responsibility for the integrity of the data and the accuracy of the data analysis. *Study concept and design*: Aning. *Acquisition of data*: Kuppanda, Hermans, Dickinson, collaborating authors. *Analysis and interpretation of data*: Kuppanda, Uren, Aning. *Drafting of the manuscript*: Kuppanda, Aning. *Critical revision of the manuscript for important intellectual content*: Uren, Cotterill, Rowe, Narahari, Dickinson, Rigby. *Obtaining funding*: Narahari, Rigby, Aning. *Supervision*: Aning.

## CONFLICT OF INTEREST STATEMENT

The authors declare no conflict of interest.

## Supporting information


**Figure S1:** Cystectomy‐Pathway Assessment Tool (C‐PAT).
Table S1: Participating centre and contributors.

## Data Availability

Data supporting the results reported in the article can be found in the BAUS C‐PAT snapshot audit dataset.

## References

[1] Stein JP , Penson DF . Invasive T1 bladder cancer: indications and rationale for radical cystectomy. BJU Int. 2008;102(3):270–275. 10.1111/j.1464-410x.2008.07743.x 18494831

[2] Liedberg F . Early complications and morbidity of radical cystectomy. Eur. Urol. Suppl. 2010;9(1):25–30. 10.1016/j.eursup.2010.01.007

[3] Yang LS , Shan BL , Shan LL , Chin P , Murray S , Ahmadi N , et al. A systematic review and meta‐analysis of quality of life outcomes after radical cystectomy for bladder cancer. Surg. Oncol. 2016;25(3):281–297. 10.1016/j.suronc.2016.05.027 27566035

[4] Edmondson AJ , Birtwistle JC , Catto JWF , Twiddy M . The patients' experience of a bladder cancer diagnosis: a systematic review of the qualitative evidence. J. Cancer Surviv. 2017;11(4):453–461. 10.1007/s11764-017-0603-6 28213769 PMC5500680

[5] Mason SJ , Downing A , Wright P , Hounsome L , Bottomley SE , Corner J , et al. Health‐related quality of life after treatment for bladder cancer in England. Br. J. Cancer. 2018;118(11):1518–1528. 10.1038/s41416-018-0084-z 29755116 PMC5988662

[6] Stangl‐Kremser J , Lambertini L , di Maida F , Martinez‐Fundichely A , Ferro M , Pradere B , et al. Enhancing recovery after major bladder cancer surgery: comprehensive review and assessment of application of the enhanced recovery after surgery guidelines. Eur. Urol. Focus. 2022;8(6):1622–1626. 10.1016/j.euf.2022.06.004 35773181

[7] Doyle C , Lennox L , Bell D . A systematic review of evidence on the links between patient experience and clinical safety and effectiveness. BMJ Open. 2013;3(1):e001570. 10.1136/bmjopen-2012-001570 PMC3549241 23293244

[8] Uren AD , Cotterill N , Abrams P , Catto JWF , Patel B , McGrath J , et al. The development of the cystectomy‐pathway assessment tool (C‐PAT): a concise tool to assess the quality of care in the cystectomy pathway. BJU Int. 2022;129(6):708–717. 10.1111/bju.15539 34218507

[9] Department of Health . Improving outcomes: a strategy for cancer. 2011. https://assets.publishing.service.gov.uk/media/5a7c5d0240f0b626628ab882/dh_123394.pdf

[10] Tamhankar AS , Thurtle D , Hampson A , El‐Taji O , Thurairaja R , Kelly JD , et al. Radical cystectomy in England from 2013 to 2019 on 12,644 patients: an analysis of national trends and comparison of surgical approaches using hospital episode statistics data. BJUI Compass. 2021;2(5):338–347. 10.1002/bco2.79 35474875 PMC8988840

[11] Russell B , Liedberg F , Khan MS , Nair R , Thurairaja R , Malde S , et al. A systematic review and meta‐analysis of delay in radical cystectomy and the effect on survival in bladder cancer patients. Eur. Urol. Oncol. 2020;3(2):239–249. 10.1016/j.euo.2019.09.008 31668714

[12] Mantle M , Dickinson A , Moody M , Cox RA . How quickly are we treating muscle invasive bladder cancer? Trends over a 17 year period. Br. J. Med. Surg. Urol. 2009;2(1):22–26. 10.1016/j.bjmsu.2008.10.002

[13] Shahid Iqbal M , Pickles R , Pedley I , et al. Delays in the diagnosis and treatment of muscle invasive bladder cancer: a pilot project mapping the pathway. J. Clin. Urol. 2015;8(4):246–251. 10.1177/2051415814557067

[14] Zhou Y , Singh H , Hamilton W , Archer S , Tan S , Brimicombe J , et al. Improving the diagnostic process for patients with possible bladder and kidney cancer: a mixed‐methods study to identify potential missed diagnostic opportunities. Br. J. Gen. Pract. 2023;73(733):e575–e585. 10.3399/BJGP.2022.0602 37253628 PMC10242858

[15] Fahmy N , Mahmud SM , Aprikian A . Delay in the surgical treatment of bladder cancer and survival: systematic review of the literature. Eur. Urol. 2006;50(6):1176–1182. 10.1016/j.eururo.2006.05.046 16846680

[16] Alessy SA , Davies E , Rawlinson J , Baker M , Lüchtenborg M . Clinical nurse specialists and survival in patients with cancer: the UK National Cancer Experience Survey. BMJ Support. Palliat. Care. 2022;14(e1):e1208–e1224. 10.1136/bmjspcare-2021-003445 35450864

[17] Kerr H , Donovan M , McSorley O . Evaluation of the role of the clinical nurse specialist in cancer care: an integrative literature review. Eur. J. Cancer Care. 2021;30(3):e13415. 10.1111/ecc.13415 33501707

[18] NICE . Access to a clinical nurse specialist. NICE Guidance. Published December 17, 2015. Accessed January 11, 2024. https://www.nice.org.uk/guidance/qs106/chapter/quality-statement-3-access-to-a-clinical-nurse-specialist

[19] Bristol Myers Squibb (BMS) . (2022). Bladder cancer patient experiences information from the 2019 National Cancer Patient Experience Survey. Fight Bladder Cancer Published February 2022. Accessed January 9, 2024. https://fightbladdercancer.co.uk/sites/default/files/downloads/Cancer_Patient_Experience_Survey.pdf

