# Supplementary material for: National audit of patient reported experience of radical cystectomy for bladder cancer pathways
Source: BJUI Compass. 2024 Aug 19;5(10):961–8. doi: 10.1002/bco2.422 (PMC11479807; doi:10.1002/bco2.422)
Supplement: Supplementary file 1 — Figure S1: Cystectomy‐Pathway Assessment Tool (C‐PAT). Table S1: Participating centre and contributors. [file BCO2-5-961-s001.docx]

**Supplementary Figure 1: Cystectomy-Pathway Assessment Tool (C-PAT)**


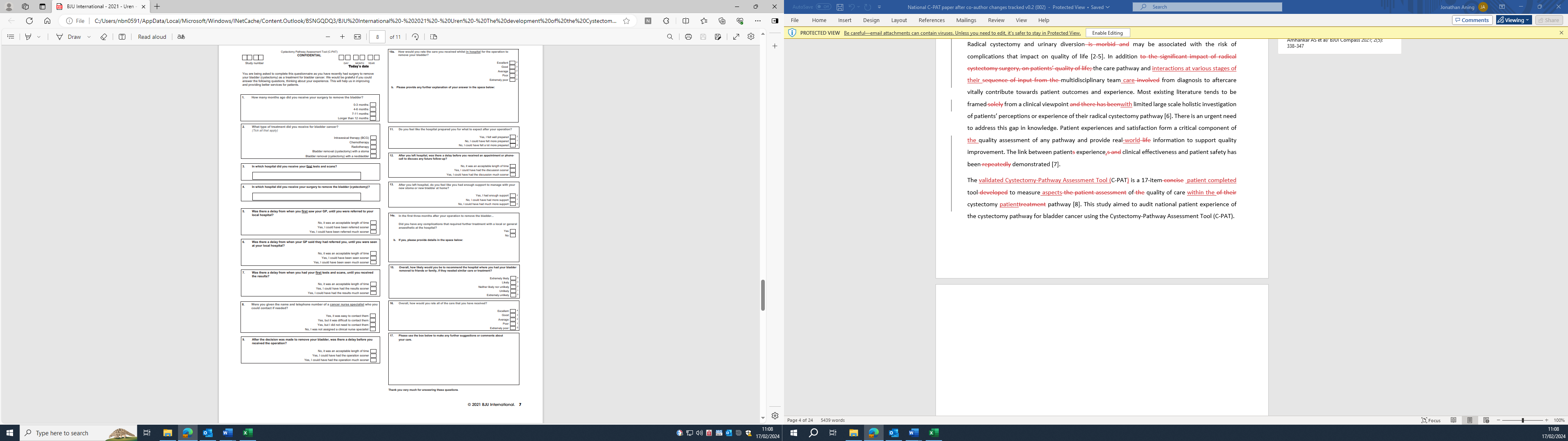


**Supplementary Table 1: Participating centre and contributors**

| Centre | Contributors |
| --- | --- |
| East Sussex Healthcare NHS Trust | Edward Calleja, Nicola Milton, Ghassan Al kefeiri, Jasmine Derex Briggs |
| Royal Surrey NHS Foundation Trust | Matthew Perry |
| Ysbyty Gwynedd Hospital, Bangor | Mohamed Abdulmajed, Ashok Bhuvanagiri, Ashok Kailasa |
| Southend University Hospital | Daben Dawam |
| East Kent Hospitals University Foundation Trust | Rustam Karanjia, Edward Streeter |
| Royal Devon & Exeter Hospital | Joseph John, John McGrath, Elizabeth Waine |
| Addenbrooke's Hospital, Cambridge | Alexandra Colquhoun |
| Western General Hospital, Edinburgh | Paramananthan Mariappan |
| University Hospital of Wales, Cardiff | Jon Featherstone, Kirthana Babureddy, Krishna Narahari |
| Nottingham City Hospital | Philip Goodall, Karen Moore, Will Green |
| New Cross Hospital, Wolverhampton | Pete Cooke |
| East and North Hertfordshire NHS Trust | Nikhil Vasdev |
| Royal Hallamshire Hospital, Sheffield | Richard Binney, James Catto |
| Stepping Hill Hospital, Stockport | Paul Cleaveland |
| University Hospitals Plymouth NHS Trust | Oliver Fuge |
| University Hospitals of Leicester | Sanya Caratella, Benjamin Jackson, Bau Valentine, Ghorab Omar |
| Southmead Hospital Bristol | Emmanuel Okpii, Jonathan Aning, Edward Rowe, Raj Pal, Anthony Koupparis |
| Christie Hospital | Vijay Sangar, Helen Johnson |
| Manchester Royal Infirmary | Vijay Sangar, Amar Mohee, Leony Gillott |
| Royal Berkshire Hospital, Reading | Mohammad Zaynulabedin Miah, Philip Charlesworth, Dipanwita Majumdar |
| Newcastle (Freeman Hospital) | Arjun Nambiar, Mark Johnson, James Leighton |
| Churchill Hospital, Oxford | Francisco Lopez |
| Gloucestershire Hospitals NHS Foundation Trust | Biral Patel, Fiona Trigg |
| Castle Hill Hospital, Hull | Matthew Simms |
| Aberdeen Royal Infirmary | Justine Royle, Deborah Munro, Morven Paterson |
| University Hospitals Coventry and Warwickshire NHS Trust | Omer Altan |
| Southampton | James Douglas |
| Royal Bournemouth General Hospital | Amit Mevcha |
| James Cook University Hospital, Middlesbrough | Jo Cresswell, Pragnitha Chitteti |
| Royal Preston Hospital (Lancashire Teaching Hospital) | Rosie Blades |
| Norfolk & Norwich University Hospital | Omar Al Kadhi |
| University College Hospital, London | Ashwin Sridhar, Anthony Ta, Kay Boyer, Hilary Baker |
| Royal Liverpool University Hospital | Vishwanath Hanchanale |
